# Sigmoid Sinus Characteristics Correlate with Early Clinical and Imaging Surrogates in Anterior Circulation Ischemic Stroke

**DOI:** 10.1007/s12035-016-0091-0

**Published:** 2016-09-10

**Authors:** Slaven Pikija, Jozef Magdic, David S. Liebeskind, Arthur Karamyan, Nele Bubel, Mark R. McCoy, Johann Sellner

**Affiliations:** 1 Department of Neurology, Christian Doppler Medical Center, Paracelsus Medical University, Salzburg, Austria; 2 Department of Neurology, Univerzitetni Klinični Center Maribor, Maribor, Slovenia; 3 Neurovascular Imaging Research Core and UCLA Stroke Center, University of California, Los Angeles, CA USA; 4 Division of Neuroradiology, Christian Doppler Medical Center, Paracelsus Medical University Salzburg, Salzburg, Austria; 5 Department of Neurology, Klinikum rechts der Isar, Technische Universität München, München, Germany

**Keywords:** Ischemic stroke, Cerebral veins, Biomarker, Densitometry, Outcome, Computed tomography

## Abstract

Cerebral venous outflow may play a decisive role in acute ischemic stroke. Here, we assessed the relation of cerebral sinus vein characteristics with clinical and imaging surrogates of early outcome in acute ischemic stroke. We evaluated cerebral vein characteristics in 212 patients with the middle cerebral artery (MCA) occlusive stroke confirmed by CT angiography CTA within 6 h from symptom onset. Readout parameters included volume and density of the sigmoid sinus (SS) and density of the superior sagittal sinus (SupSagS). These were correlated with early clinical outcome defined as hospital death (HD), final infarct volume (FIV), and National Institute of Health Stroke Scale (NIHSS) at discharge. We found a correlation for the volume of the right SS and the FIV when the M1 segment of the MCA of either side was occluded (*p* = 0.002, Rho = 0.206, *n* = 134). A decrease in SS density was more pronounced in the subgroup with unfavorable outcome (NIHSS > 15 + HD) but only when the left hemisphere was affected (*p* = 0.026, *n* = 101). On stepwise logistic regression analysis, adjusted for on-admission NIHSS, age at presentation, and FIV, smaller SS volume was independently associated with lower odds for hospital death (*n* = 183, OR 0.13, 95 % CI 0.02–0.94, *p* = 0.043). A larger right SS and a decrease in density increase the risk of unfavorable early clinical and imaging outcome in AIS. This finding of an outflow pattern independent of the stroke site implicates an involvement of the cerebral venous drainage system in the pathophysiology of ischemic stroke.

## Introduction

Recent advances in vascular neuroimaging in patients with acute ischemic stroke (AIS) have led to the identification of prognostic factors. Determinants of outcome include the presence and density of a hyperdense artery sign, and location and extent of proximal artery occlusion [1, 2]. There is also emerging evidence that good collateral circulation during AIS is associated with favorable clinical outcome, lesser extent of infarct volume, and success of revascularization measures [3–6]. The influence of venous structures and the basal venous sinuses, however, has been evaluated only scarcely in this context. Since the majority of the cerebral blood volume in the brain is contained in the venous pool, a restricted outflow may be of prognostic relevance. A recent study suggested that limitation of the ipsilateral venous drainage could contribute to development of edema in the setting of malignant middle cerebral artery (MCA) infarction [7]. Cortical venous drainage may also play a prognostic role for clinical outcome [8].

The great veins are interconnected and valveless, with the theoretical possibility of bidirectional blood flow. Blood from both cerebral hemispheres is drained mainly via the superior sagittal sinus into the confluence of sinuses and then via the transverse and sigmoid sinuses into the jugular bulb on the each side. A variable portion of the superficial temporal cortex is an exclusion and drains to the cavernous sinus and vein of Labbé to the ipsilateral distal or transversal sinus. However, this concept is challenged by significant anatomical variability and various extent of atresia [9, 10]. Whether the abnormalities of the venous system morphology are analogous to the consequences of an incomplete Willis circle has not been investigated so far [11]. The sigmoid sinus is known to be largely void of arachnoid granulations, thus allowing mostly unbiased analysis of its volume and density in simple axial plane on nonenhanced and enhanced CT images [12]. Here, we investigated a potential relationship of cerebral sinus vein characteristics with clinical and imaging surrogates of early outcome in acute ischemic stroke (AIS).

## Subjects and Methods

We conducted this retrospective study at two Central European stroke centers. These were the Christian Doppler Medical Center in Salzburg, Austria (CDK) and the University Medical Centre in Maribor, Slovenia (MB). Only patients with CT angiography (CTA)-confirmed occlusion of M1 or M2 segment of the MCA were included. We excluded patients with CT scan performed more than 6 h after symptom onset, patients with unknown symptom onset time, and patients with additional pathologies such as brain tumors, arteriovenous malformations, and traumatic brain injury. Also excluded were patients with CT scans of low/unreadable quality. All data acquisition was done by purely retrospective chart review. Subsequent analyses were performed on anonymized data and therefore institutional review board approval was waived due to national regulations.

We recorded symptom-to-CT and time-to-control CT time points. NECT and CTA angiography scans were acquired at CDK with multidetector CT scanner Sensation 64 (Siemens, Erlangen, Germany) and in MB with multidetector CT scanner Aquilion 64 (Toshiba Medical Systems, Otawara, Japan). The NECT scans were reconstructed into 4-mm-thick adjacent slices through the whole brain. For CTA aquisition in MB and CDK 60 ml Iomepron contrast agent was injected at a rate of 5 ml/s in MB and 4 ml/s in CDK and scanning would be triggered when contrast density in the arch of aorta was 180 HU in MB and 100 HU in CDK. Scanning time was set to 4-7 s in MB and 3 sec in CDK﻿. Axial NECT and CTA images were analyzed with the IMPAX tool. Evaluation of CT scans was performed at each site by experienced stroke physicians blinded to the clinical data.

### Institutional Standard Procedure with Acute Stroke Patients

All patients underwent a stroke diagnostic pathway including brain CT diagnostics (NECT and CTA), blood examinations focusing on the identification of stroke etiology, transthoracal echocardiography, and 24-h ECG monitoring. Intravenous thrombolysis and mechanical thrombectomy were performed according to institutional guidelines.

### Quantification of the Superior Sagittal and Sigmoid Sinus Volume and Density

**Fig. 1:**
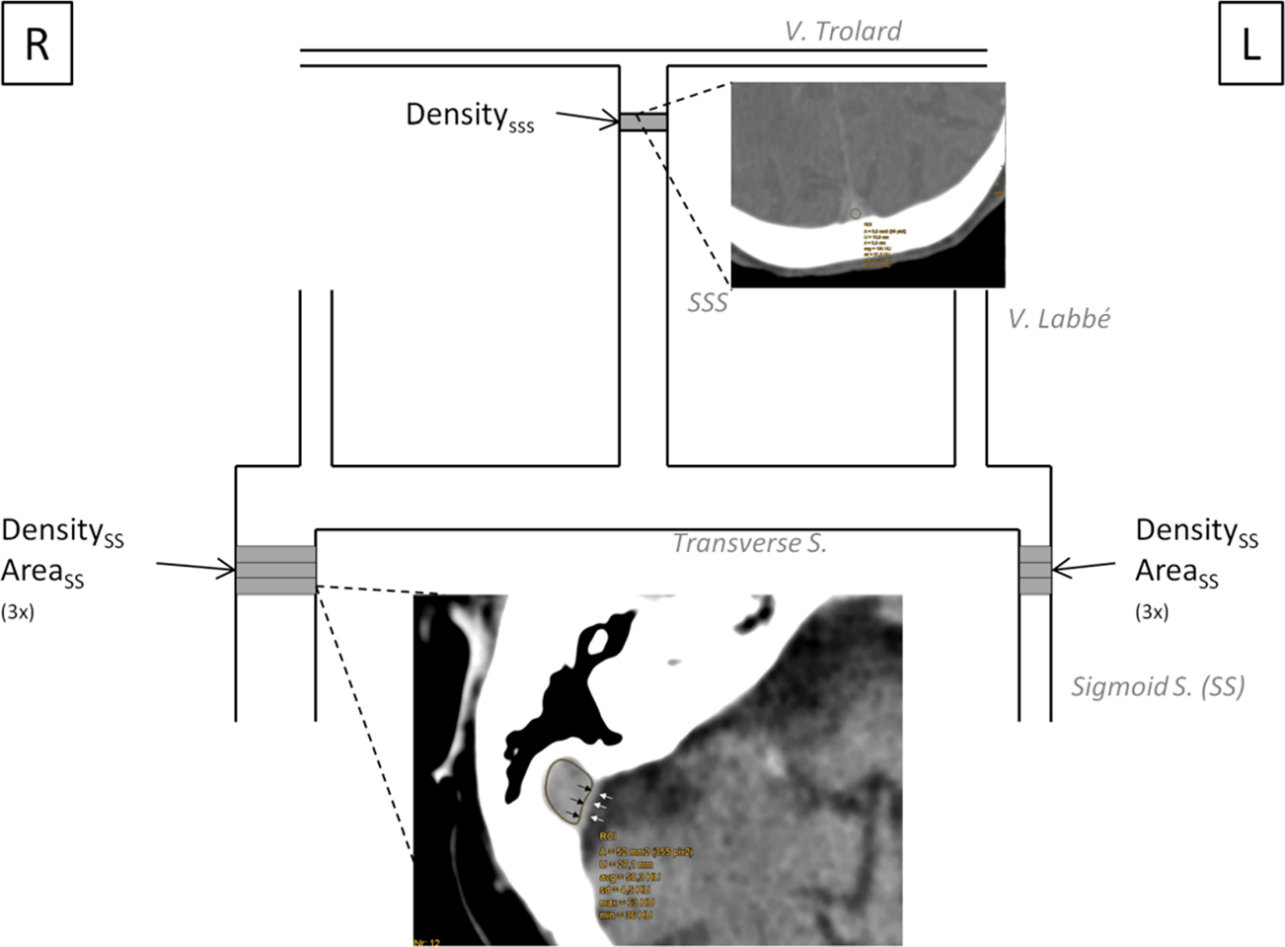
Schematic diagram of the cerebral venous system and the position of regions of interests (ROI) in nonenhanced brain CT (NECT) and CTA. In a first step, the superior sagittal sinus (SSS) average Hounsfield units (avg) values were recorded using the CT-A image. In the next step, a ROI is placed within the right sigmoid sinus on NECT. The bony part and the medial dural wall are avoided (*black* and *white arrows*). The A (area) in square millimeters and avg values are recorded. This procedure is repeated three times (3×) to calculate sigmoid sinus volume in cubic millimeters and average density. *R* right, *L* left side

The quantification of sinus volume and density is described in Fig. 1. Briefly, a round region of interest (ROI) was placed in the superior sagittal sinus (SupSagS) just above the lateral ventricles, and measurement of the average density in Hounsfield units were recorded (HU). We identified the right and left sigmoid sinuses (SS). The “freehand” ROI was placed inside the SS with the attention not to involve the bone and dural parts. Area and density measurements were then made on next two consecutive sections caudally on NECT and on the each second of the sections on CTA—due to thinner slices. Finally, the volume of the SS in cubic millimeters was determined by multiplying the measured areas with the distance between two slices. Validation experiments confirmed high interrater reliability of the method. Volumes of both SS were summed to calculate the summed SS volume (SUMSSV). Dominance of the sigmoid sinus was arbitrarily defined as the volume of the SS equal or larger than 80 mm^3^ from the opposite side.

### Derived Density of Sigmoid Sinuses Using CTA

For CTA, we measured the density in the jugular bulb on both sides. The density of SupSagS was multiplied by 2 and then subtracted from sum of both SS densities in order to derive the calculated density of both SS (CDSS).

### SS Volume Ratio

We calculated the SS volume ratio as follows: right SS volume/left SS volume. When the right SS was atretic, the ratio was given an arbitrary value of 0.01. When the left SS was atretic, the ratio was given a value of 20.

### Difference in Density Between SupSagS and Each SS-SSDelta

**Fig. 2:**
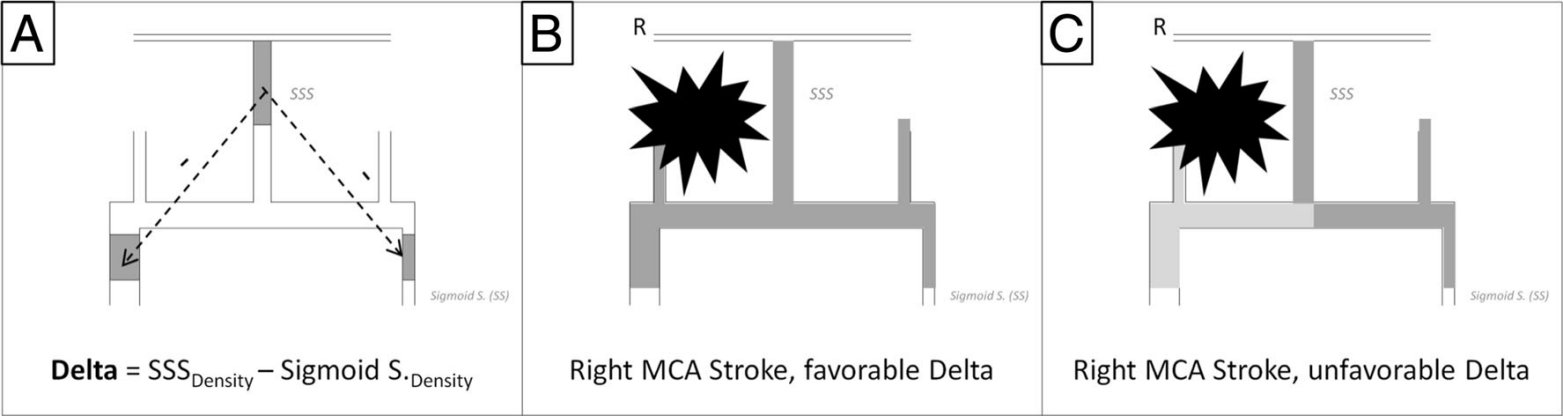
Densitometry of sigmoid sinuses. Measurements of delta density in between SSS and sigmoid sinus (**a**). Delta negativity—in the setting of right middle cerebral artery stroke (MCA), there is no decrease of density in right sigmoid sinus (favorable situation) (**b**). Unfavorable outcome, however, is associated with sigmoid sinus density decrease in respect to SSS density (**c**). *SSS* superior sagittal sinus

In order to account for density differences between superior sagittal sinus and sigmoid sinus as a sign of possible outflow impairment, we calculated SSDelta value as SupSagS HU density − SS HU density separately for each side. However, SS could be atretic (nonexistent); in this case, SSDelta would be arbitrarily given the density value of SupSagS. With regard to the affected hemisphere, we considered two possibilities with regard to SSDelta value. The first option was when SSDelta on ipsilateral site was negative. This pattern was considered favorable since the ipsilateral sinus density was not lower than SupSagS. The second situation was when SSDelta on ipsilateral site was positive. This condition was considered unfavorable since the ipsilateral sinus density was not lower than SupSagS (Fig. 2).

### Quantification of the Final Infarct Volume

The follow-up CT scans performed more than 24 h and less than 7 days were examined for presence of infarction, when present. The hypodense area was manually delineated on each CT slice (4-mm height) which produced the area in square centimeter. Finally, the volume in cubic centimeter was calculated from the measured area and the corresponding slice thickness.

### Quantification of Symptom Severity

The National Institutes of Health Stroke Scale (NIHSS) scoring on admission and transfer were available in the patient charts. Unfavorable outcome was defined as NIHSS > 15 and hospital death (HD).

### Data Analysis

Data about patients’ demographics, medical history, imaging features, and clinical outcome were evaluated with descriptive statistics. Categorical variables were compared using Fisher’s exact or *χ* ^2^ test, while continuous variables were compared using the Mann-Whitney test for independent samples. The association between the variables was assessed by rank correlation. The relationship between different variables was analyzed by using multiple regression. In order to compare interrater HU and area measurement (reliabilities), we use intraclass correlation coefficient (ICC).

All statistical analyses were performed using the STATA 13.0 software 2 (Science Plus Group, Groningen, The Netherlands).

## Results

In Salzburg, we screened 2833 CT scans of consecutive patients with AIS admitted to CDK between June 2012 and December 2015 and identified 179 patients. In Maribor, 33 patients could be found by systematic search among 1796 consecutive patients with AIS in the period of January 2011 to January 2015. Among a total of 212 patients, CTA images were of sufficient quality in 197 cases (92.9 %). Patient demographics, treatment, and outcome are presented in Table 1.

**Table 1:** Patient characteristics (*n* = 212)

Characteristics (*n*)*	Values
Demographics (212)	
Age (years)	72 (63, 84)
Women	120 (56.6)
Medical history (212)	
Prior stroke/TIA	31 (14)
Atrial fibrillation	107 (50)
Peripheral artery disease	12 (6)
Carotid stenosis >50 %	31 (14)
Arterial hypertension	149 (70)
Diabetes mellitus	32 (15)
Chronic heart failure	40 (19)
Use of antiplatelets^†^	57 (27)
Use of anticoagulants	28 (13)
Stroke type by TOAST (212)	
Cardioembolic	115 (54)
Unknown	52 (25)
Undetermined	5 (2)
Other	5 (2)
Clinical presentation	
NIHSS (points) (212)	16 (11,20)
Serum glucose (mmol/L) (210)	118.8 (106.0, 141.0)
Erythrocytes (×10^12^/L) (179)	4.5 (4.1, 4.8)
HbA1c (mmol/L) (159)	5.6 (5.3, 5.9)
Fibrinogen (mg/dL) (208)	355.5 (306.0, 436.0)
C-reactive protein (mg/L) (159)	0.4 (0.18, 1.0)
Acute treatment (212)	
Thrombolysis (rt-PA)	156 (74)
Thrombectomy	107 (50)
Thrombolysis + thrombectomy	86 (41)
Thrombectomy outcome [TICI] (102)	
No perfusion [0]	18 (18)
Penetration, no distal filling [1]	3 (3)
Perfusion, <50 % distal filling [2a]	7 (7)
Inadequate [0–2a total]	28 (28)
Perfusion, >50 % distal filling [2b]	20 (20)
Full perfusion [3]	54 (53)
Adequate [2b–3 total]	64 (63)
Imaging particulars	
Affected vessel (212)	
Middle cerebral artery M1	154 (73)
Middle cerebral artery M2	58 (27)
Final infarct volume (cm^3^) (197)	41.2 (9.9, 134.1)
Control image finding (201)	
Infarction	154 (77)
Hemorrhagic infarction	33 (16)
Resolution (infarct volume = 0)	14 (7)
In-hospital mortality (212)	40 (19)
NIHSS at discharge (points) (172)	7.5 (4, 15)

Data are presented as median (25, 75 percentile) or count (percent)

*HbA1c* glycated hemoglobin, *NIHSS* National Institutes of Health Stroke Scale, *rt-PA* recombinant human tissue plasminogen activator, *TIA* transitory ischemic attack, *TICI* thrombolysis in cerebral infarction grading, *TOAST* Trial of Org 10172 in Acute Stroke Treatment

The anatomical evaluation for different cerebral sinuses is shown in Table 2. SS volume (when measured) on the right and left sides showed a clear correlation between NECT and CTA measurements (*p* < 0.001 for both). The right SS was more often found to be present on CTA than on NECT (*n* = 206, 97.1 % and *n* = 197, 98.5 %, respectively). The left SS was detected on CT in 90.8 % (*N* = 206) and on CTA in 93.9 % (*N* = 197). The frequency of the atretic SS on CTA or NECT did not show side preferences in relation to the affected hemisphere.

**Table 2:** Measurement of cerebral venous sinuses with nonenhanced CT and CT angiography (*n* = 212)

Modality	CT		Modality	CT angiography	
Sinus/vein	Volume (mm^3^)	Density in HU	Sinus/vein	Volume (mm^3^)	Density in HU
Sigmoid right (200)	305.0 (191.4, 392.8)	50.4 (47.0, 55.2)	Sigmoid right (194)	357.4 (239.6, 470.4)	131.8 (99.7, 171.8)
Sigmoid left (187)	212.4 (136.0, 299.2)	51.1 (47.0, 56.2)	Sigmoid left (185)	277.2 (160.0, 375.6)	141.7 (104.5, 176.0)
Sagittal sinus (210)	–	56.6 (51.4, 60.5)	Sagittal sinus (199)	–	176.0 (132.0, 215.0)
–	–	–	Jugular bulb right (188)	–	102.0 (77.5, 141.0)
–	–	–	Jugular bulb left (172)	–	110.0 (74.8, 143.0)

Data are presented as median (25, 75 percentile) or count (percent)

*HU* Hounsfield units

### NECT

The right SS was dominant in 52.4 % and the left SS in 22.3 %. We noted codominance in 25.2 %. The final infarct volume did not differ between sex (*p* = 0.19) and had no correlation with the patients’ age. Women had significant lower total SS volume than men (median 466.8 and 562.8 cm^3^, respectively; *p* < 0.001).

### CTA

The right SS was dominant in 50.8 %, the left SS in 25.9 % and they were codominant in 23.3 %. We found a negative correlation of CDSS and SUMSSV, indicating that the larger sinuses had less contrast enhancement (*p* = 0.002, Rho = −0.227). Women had significantly lower SumSSV (median 692.0 vs. 566.8 cm^3^, *p* < 0.001). The cases in the lowest quartile of SUMSSV (first 25 %, *n* = 50, 25.4 %) tended to be younger. However, this observation did not reach statistical significance (*p* = 0.063).

### Interrater Agreement

Interrater agreement for the NECT-measured venous densities in HU and areas of SS was determined in 20 randomly selected patients. The evaluation was performed by three raters for NECT (SP, AK, NB) and by two raters (SP, NB) for CTA. The agreement for SupSagS density as measured on NECT was excellent: ICC = 0.88, for density and area of right SS ICC = 0.92 and ICC = 0.85, respectively, and for density and area of left SS ICC = 0.98 and ICC = 0.79, respectively. On CTA, the SupSagS density correlation was good (ICC = 0.77). The ICC for the density of the right SS and area of the right SS were ICC = 0.90 and ICC = 0.77, respectively. The density and area of the left SS ICCs were ICC = 0.95 and ICC = 0.95, respectively.

### Volumetric Measurements

#### CTA and Clinical Outcome

Patients in the lowest quartile of combined SS volume (*n* = 50) when compared with others had less HD (6.0 and 22.4 %, *p* = 0.009) and less unfavorable outcome (22.0 vs. 43.5 %, *p* = 0.007). When the left SS was dominant, the risk of hospital death was higher when the ipsilateral (left) hemisphere was affected (*n* = 101, *p* = 0.017). The side of atresia (or volume below 5 %) in respect to the affected hemisphere had no relation to clinical outcome.

#### CTA and Imaging Outcome

**Fig. 3:**
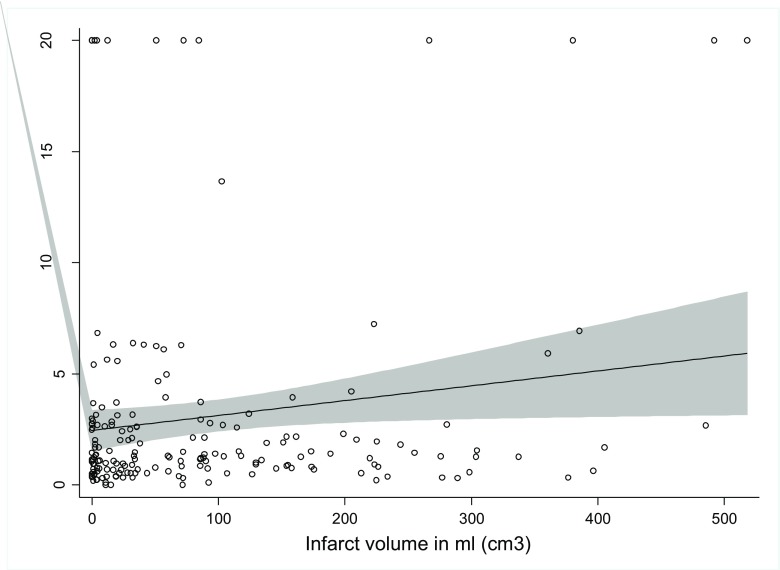
Correlation of right sinus sigmoideus (SS) volume, expressed as ratio between the right and left SS volume, with final infarct volume in 183 acute stroke patients, *p* = 0.036

We identified a correlation for right SS volume and the FIV when the M1 segment of either side was occluded (*p* = 0.002, Rho = 0.206, *n* = 134). This association was even more pronounced when the left MCA M1 was occluded (*p* = 0.036, Rho = 0.259, *n* = 66) and weaker for the right-sided M1 occlusion (*p* = 0.159, *n* = 68). The left SS volume did not show a correlation with FIV. We detected an impact of the left SS volume on FIV when the ratios of the SS volumes were analyzed. The ratio of the right SS volume divided by the left SS volume (rSS volume/lSS volume) showed correlation with FIV (*n* = 183, *p* = 0.036), as shown in Fig. 3.

#### CT and Imaging Outcome

The volume of the right SS was positively correlated with the FIV when MCA M1 of either side was occluded (*p* = 0.046, Rho = 0.169, *n* = 139). This positive association remained present when right MCA M1 was occluded at (*p* = 0.028, Rho = 0.259, *n* = 72). Yet, no such correlation was found for the left MCA M1 occlusion (*p* = 0.159, *n* = 68). No association for the left SS volume and FIV was found.

### Densitometric Measurements

#### CTA and Clinical Outcome

We found a trend for the correlation of the density of the right SS and unfavorable outcome in the ipsilateral MCA M1 occlusion (*p* = 0.051). There was also a trend for SSDelta negativity (the ipsilateral SS sinus was more dense than SupSag) and favorable outcome (*p* = 0.055).

#### CTA and Imaging Outcome

The density of the right SS correlated with FIV in the setting of the ipsilateral M1 occlusion (*p* = 0.036). The left SS volume did not show a correlation with FIV.

### Multivariate Analysis

The smaller volume of both SS (SUMSSV) as measured on CTA was significantly associated with less odds for hospital death (*n* = 183, OR 0.13, 95 % CI 0.02–0.94, *p* = 0.043) on stepwise logistic regression analysis, including on-admission NIHSS, age at presentation, and FIV. When FIV was excluded from the analysis, the SS volume was associated with smaller odds for unfavorable clinical outcome (*n* = 197, OR 0.32, 95 % CI 0.12–0.85, *p* = 0.023).

## Discussion

These novel findings establish the potential role and impact of the structural status of the cerebral venous system in anterior circulation AIS. The volumetric analysis of the SS revealed univariate and multivariate associations with early clinical and imaging outcomes. In this regard, our study disclosed that a larger right SS in terms of pronounced asymmetry is associated with hospital death and the larger final infarct volume in the setting of MCA M1 occlusion of either side. Densitometry on the other hand showed weaker associations with the outcome surrogates. In the univariate analysis, the decrease in density of the ipsilateral sigmoid sinus to the MCA infarct showed a trend for unfavorable outcome. In the univariate analysis, the right SS density showed a negative association with the FIV. Thus, we propose a potential role of venous outflow in the pathophysiology of ischemic stroke.

The findings of this study extend the previous work that showed significance of impaired cortical venous outflow as measured on the CTA on prognosis in acute stroke [8]. It was established that density, especially those of the superficial intracerebral veins, predicts late outcome (90 day) even when adjusted for baseline NIHSS score. In our sample, the density of the right SS correlated with FIV when the ipsilateral hemisphere was affected.

It is known that atresia of one of the sinuses could lead to impaired venous outflow, thus worsening brain edema and leading to unfavorable outcome [7]. However, reports in this direction are only available on small number of patients and results were positive only for atresia/thrombosis ipsilateral to affected hemisphere. In our series, we could not confirm these findings for early clinical or imaging outcomes and pure or near-atresia (defined as the volume of SS under 5 % of the group).

As proposed by Liebeskind et al., the elevated pressure in venous system could provide heightened downstream resistance for therapeutic flow augmentation [13]. This is corroborated indirectly by our study showing that lower density of SS translates to worse outcome. We can consider ipsilateral density as a surrogate marker for heightened venous pressure. Notably, in our study, only 4/21 (19 %) cases without decrease in SS density had unfavorable clinical outcome.

Dural sinuses and deep cerebral veins are capable to reverse flow in physiologic situations mainly in elderly population [14]. This possibility could provide compensatory outflow resistance via retrograde flow thus offsetting the collapse of the cerebral veins and potentially eliminating blood flow maldistribution [15]. There is a possibility as suggested by our work that the disproportionality between SSs could impair such possible collateralization.

The proposed measurements of the SS characteristics are fast and practical and could be done within 5 min. Moreover, it is highly reproducible as shown by interrater correlation data. The limitations of the current study are few but important. The sample is limited only to the cases with performed CTA, thus biasing the population to the more affected individuals where this examination was judged as necessary. However, the study was performed in two university centers, thus reducing additional selection bias. Moreover, we did not account for other accessory venous outflow patterns such as cervical epidural venous system through anterior condylar confluent system. However, this is a rare constellation [16, 17]. Furthermore, we used CTA technique that is not time-resolved, and as the consequence, the measured density of the venous compartment decreases from sagittal sinus to jugular bulb. In this regard, we needed to account for atresic sinuses. Regardless, we sought to determine the density of the sigmoid sinus in order to extrapolate the amount of venous outflow. Eventually, future studies should evaluate the outcome beyond the acute phase, e.g., after 3 months.

In conclusion, we have extended the previous observation of impact of impaired superficial venous outflow on clinical outcome. The outflow pattern we identified for unfavorable prognosis was independent from the site of stroke and, thus, may play a decisive role in the early pathophysiology of stroke. Further studies, especially time-resolved ones reflecting directionality of the venous outflow in the setting of arterial pathology are mandatory to shed more light on this neglected but emerging theme.
